# The Facile Modification of Polyacrylate Emulsion via Hexadecane to Enhance Controlled-release Profiles of Coated Urea

**DOI:** 10.1038/s41598-018-30585-5

**Published:** 2018-08-16

**Authors:** Yazhen Shen, Changwen Du, Jianmin Zhou, Fei Ma

**Affiliations:** State Key Laboratory of Soil and Sustainable Agriculture, Institute of Soil Science, Chinese Academy of Sciences, 71N. East Beijing Road, Nanjing, 210008 China

## Abstract

Development of controlled-release urea (CRU) has attracted research attention because of food scarcity problems and environmental concerns. To slow down the nutrient release of CRU coated with waterborne polyacrylate, conventional emulsion polymerization (CEP), conventional emulsion polymerization containing hexadecane (CEP + HD), and miniemulsion polymerization (MP) were carried out to discern the influence of polymerization technique and hexadecane on the properties of emulsions, films, and on the resultant nutrient release profiles of controlled-release urea. The addition of hexadecane improved water resistance, decreased the glass-transition temperature, and slowed down the nutrient release. CEP + HD was superior to MP in retarding nutrient release since the majority of HD was distributed in the exteriors of the particles of the former and interiors of the particles of the latter. Exterior HD improved water resistance more effectively, while interior HD reduced glass-transition temperature more significantly. Overall, our findings showed that incorporation of HD into polyacrylate emulsion produces excellent coatings that delay the release of urea. It has great potential application in controlled-release fertilizers coated with waterborne polymers.

## Introduction

With rapid growth in world population, the demand for fertilizers has increased to meet the agricultural production levels needed to satisfy the global food demand^1^. Nitrogen is a necessary nutrient for plant growth and an important factor in crop production^2^. On the global scale, nitrogen fertilizer consumption is expected to increase to 130–150 million ton/year by 2050^3^. However, low efficiency of nitrogen fertilizer (about 30–50%) due to losses via runoff, leaching, and volatilization contributes to the eutrophication of water bodies and the greenhouse effect via emissions of nitrogen-containing gases^4,5^. Therefore, development of controlled-release urea (CRU) with high efficiency is significant for a sustainable agricultural system.

The coating material used to produce CRU controls the nitrogen release rate. Polymers are widely used as coating materials because the nutrient release of polymer-coated fertilizers is sustainable and predictable^6^. The coating polymer comprised polymers that are soluble in organic and aqueous solvents. Nowadays, more and more attention has been paid to the aqueous polymers due to their benign environmental and safety advantages^7–11^. Among the many aqueous materials, aqueous polyacrylate has been widely adopted because of its excellent film-forming properties, appropriate viscosity, and low price^12^. Although it is degraded at a relatively low rate, it is also environmentally friendly because it is non-toxic and does not cause eutrophication^13^. It also increased soil microbial metabolic activity without adverse effects on soil bacterial community composition and soil structure^14,15^. In addition, it was used as drug carrier for targeted delivery towards cancer treatment *in vivo*^16^. Therefore, it can be seen that is safety for animals and humans. However, low tolerance of aqueous polyacrylate to water permits CRU to release excessive urea during the first several days. This can easily burn seedlings when CRU and conventional urea are commingled in a single application^17^. The low tolerance to water also reduces the nutrient release duration of CRU and causes imbalance between the crop’s long-term requirement and the short nutrient release longevity (often <30 d)^18^. The drawbacks limit the large-scale use of polyacrylate-coated urea in the field, particularly with respect to crops with relatively long growing periods. Thus, it is necessary to significantly improve the nutrient release characteristics of aqueous polyacrylate-coated urea.

In our previous studies, an aziridine crosslinker and Fe^III^-tannic acid complexes were used to modify aqueous polyacrylate, and the resultant release profiles of coated urea exhibited satisfactory improvement^19,20^. Additionally, miniemulsion polymerization (MP) has been proved beneficial over conventional emulsion polymerization (CEP) in retarding nutrient release^21,22^. MP involves the use of a highly hydrophobic co-stabilizer, appropriate surfactant, and homogenization devices to produce nanometer-sized monomer droplets^21^. Monomer droplets in a miniemulsion are dominant sites for particle nucleation^23^. A co-stabilizer, hexadecane, is used to increase the osmotic pressure to suppress the coalescence of oil droplets caused by Laplace pressure (Ostwald ripening)^24–26^. CEP refers to aqueous macro-emulsion polymerization, and the main reaction locus in which is the nucleated monomer-swollen micelle.

Although MP has been proved beneficial over CEP in retarding nutrient release, it is not clear whether it is the miniemulsion polymerization technique or the co-stabilizer that causes slow nutrient release. Besides, an inefficient homogenization device (Ultrasonifier), instead of an efficient homogenization device (high pressure homogenizer), was used to produce miniemulsions by Shen^22^. The first goal of this study was to explore the intrinsic reason for retarding nutrient release of polyacrylate-coated fertilizer. The second goal was to confirm the preferred controlled-release profile of CRU coated with high-pressure-homogenizer-emulsified polyacrylate miniemulsion. Polyacrylate emulsions were prepared by CEP, CEP containing hexadecane (CEP + HD), and MP under different homogenizer pressures and pass numbers to investigate the influence of polymerization technique and hexadecane on the properties of the emulsions, films, and the resultant nutrient release profiles of CRU.

## Results

### The morphology of HD and polyacrylate emulsions

**Figure 1:**
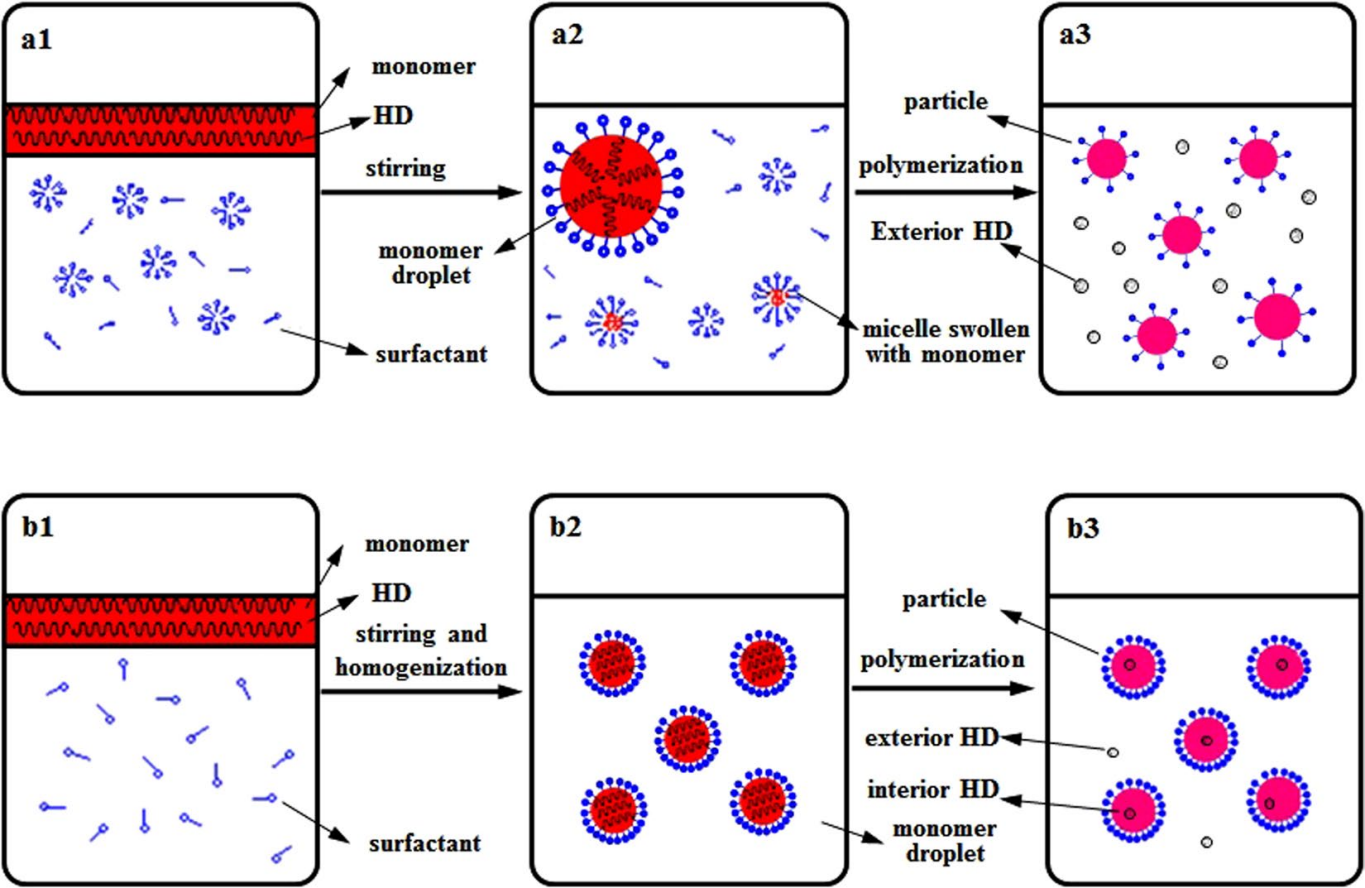
Schemes for the distribution of HD in monomer droplets and particles during the polymerization process for CEP + HD (**a**) and MP (**b**). CEP + HD represents the emulsion containing HD synthesized by conventional emulsion polymerization CEP, MP signifies miniemulsion polymerization.

**Figure 2:**
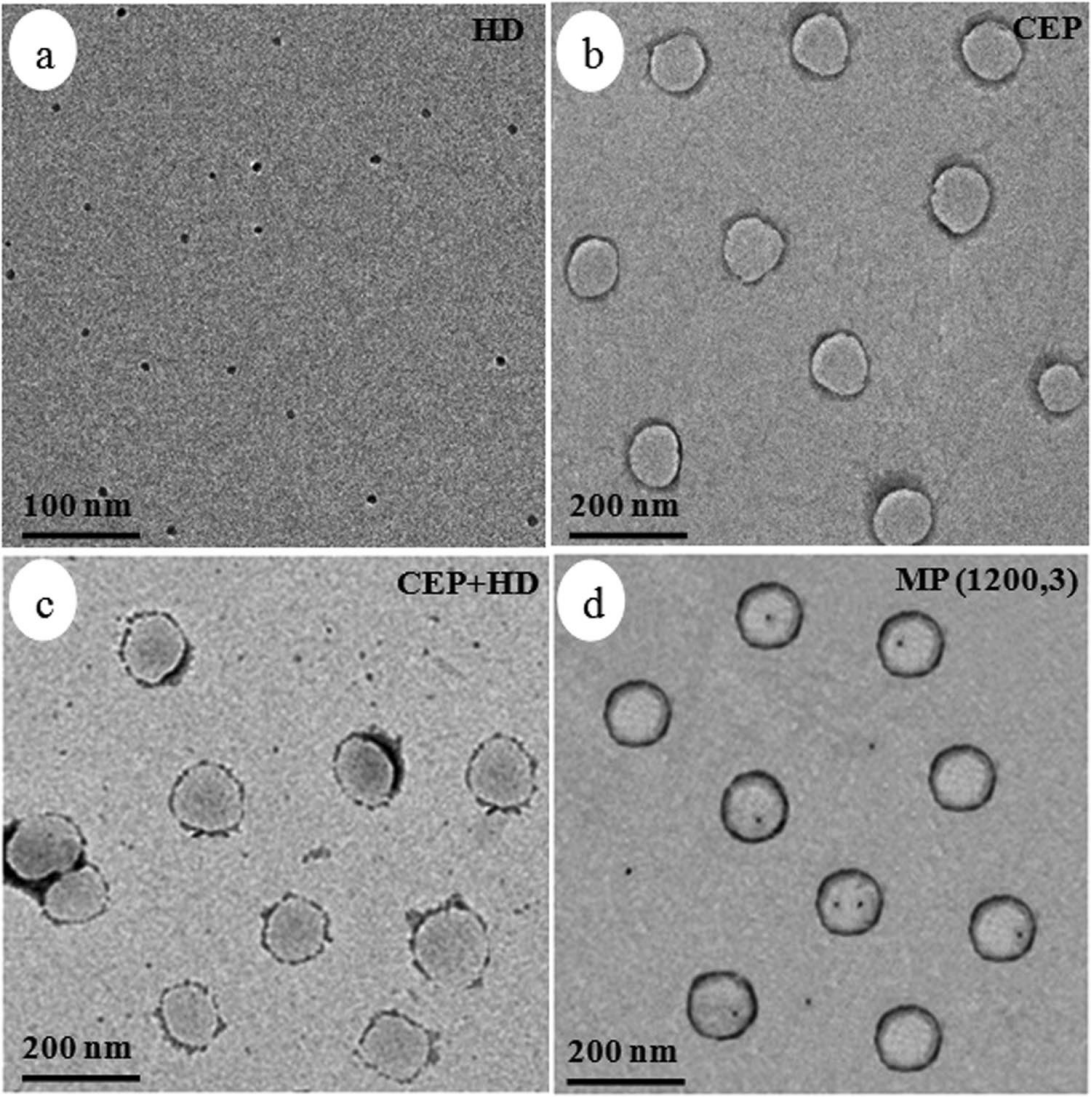
TEM images of HD (**a**) CEP (**b**) CEP + HD (**c**) MP(1200,3) (**d**). CEP denotes conventional emulsion polymerization, CEP + HD represents the emulsion containing HD synthesized by CEP, MP (1200, 3) signifies miniemulsion polymerization through homogenizer pressures of 1200 bar, and pass number of 3.

The distribution of HD in particles during polymerization was of critical importance in controlling properties of the coating. The representative distribution difference between CEP + HD and MP during polymerization is shown in Fig. 1. In CEP + HD (Fig. 1a), HD was dissolved in monomer from the beginning (a1). After stirring, HD was evenly distributed inside monomer droplets (a2). Once initiating polymerization, free radicals entered micelles, and the micelles swollen by monomers were the main loci of nucleation and polymerization. As polymerization progressed, monomer diffused from monomer droplets to micelles or particles through the aqueous phase. The hydrophobicity of HD made it difficult to transfer through the aqueous phase to particles. Therefore, the majority of HD was distributed in the exteriors of the particles and the emulsion system contained HD droplets in addition to polymer particles (a3)^27^. In MP (Fig. 1b), HD was dissolved in monomer at the start (b1) and evenly distributed inside monomer droplets after stirring and homogenization (b2) just as in CEP + HD. whereas, droplet nucleation was the predominant particle nucleation mechanism. This allowed HD to avoid passing through the aqueous phase to particles, so HD was equally distributed in the particles as that in the droplets at the beginning of the polymerization (b3). The above mechanism was substantiated by the TEM images of HD, CEP, CEP + HD and MP(1200,3) (Fig. 2). It indicated that HD was black spots and CEP particles were light spheres. The image of CEP + HD exhibited HD was distributed in the exteriors of the particles, while the majority of HD was distributed in the interiors of the particles of MP(1200,3). MP(1200,3) exhibited a uniform distribution, whereas CEP and CEP + HD exhibited a broader size distribution compared with MP(1200,3).

### Release profiles of CRU

**Figure 3:**
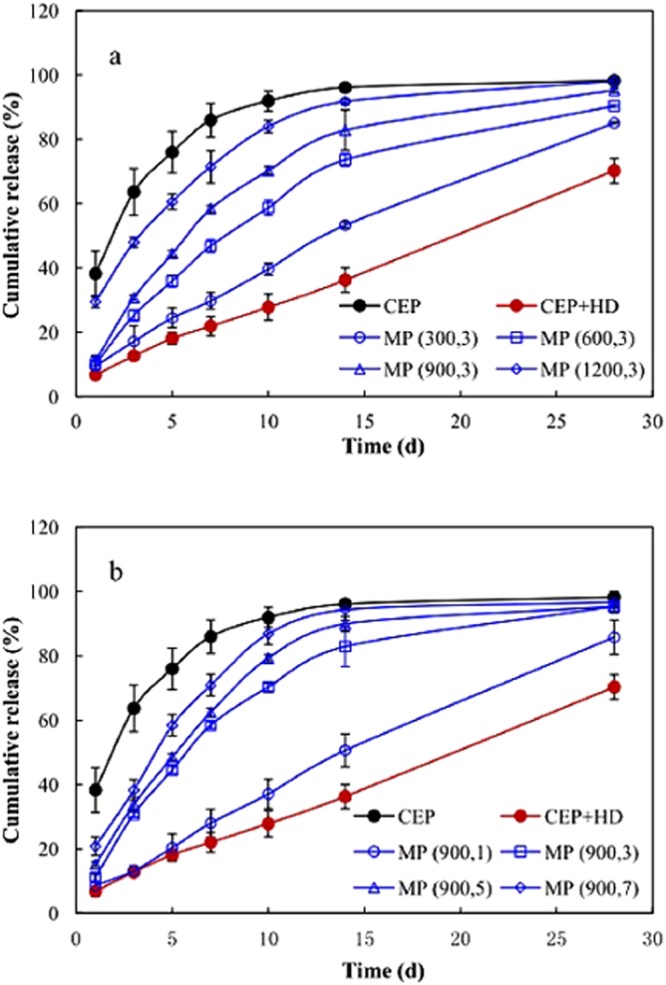
Nutrient release profiles of CRU prepared via CEP, CEP + HD and MP under different homogenizer pressure (**a**) and pass number (**b**). CEP denotes conventional emulsion polymerization, CEP + HD represents the emulsion containing HD synthesized by CEP, MP (m, n) signifies miniemulsion

Nutrient release profile was essential to evaluate whether the coating was suitable for CRU. The nutrient release profiles of CRU prepared via CEP, CEP + HD, and MP at different homogenizer pressures and pass numbers are displayed in Fig. 3. The incorporation of HD into CEP (CEP + HD) exhibited strikingly delayed release from CRU in comparison with CEP. Firstly, the preliminary solubility of CRU decreased from 38.3% (CEP) to 6.8% (CEP + HD). The high preliminary solubility for CEP could easily burn seedlings when CRU and conventional urea are commingled in a single application. Secondly, the cumulative release curves changed from an “inverted-L” shape (the nutrient release went up rapidly in the beginning and then leveled off) to a “linear” shape (the nutrient released gradually during the entire period) after the incorporation of HD into CEP. Plant growth requires a sustainable supply of nutrients, thus CRU coated with CEP + HD supplies available urea synchronously with sequential plant needs. MP also exhibited slower urea release than CEP and the slowing extent decreased with increase in homogenizer pressure (Fig. 3a) and pass number (Fig. 3b). These results illustrated that the release rate of urea could be tailored by controlling homogenizer pressure and pass number during the preparation of MP. The inferior performance of MP relative to that of CEP + HD in retarding urea release indicated that the slower urea release of CRU coated with MP than that with CEP was mainly associated with HD in the recipe of the miniemulsion, rather than the miniemulsion polymerization technique.

### Particle size distribution profiles

**Figure 4:**
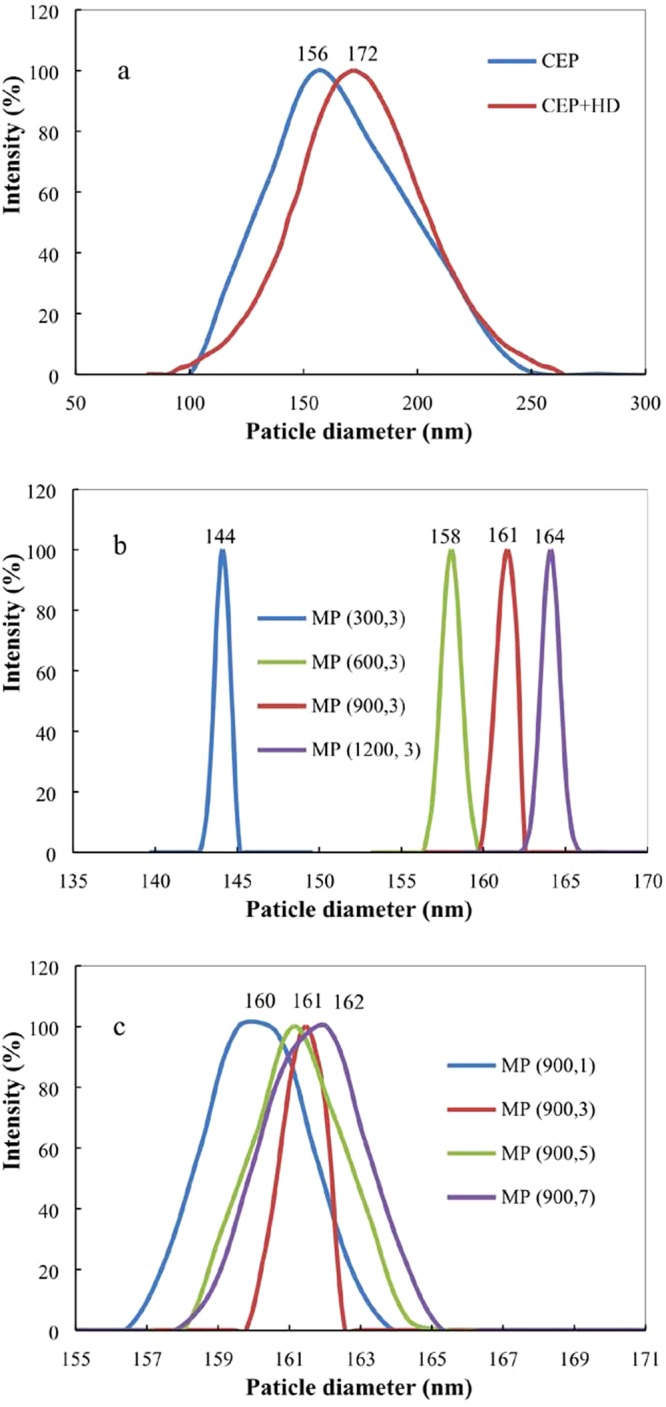
Particle size and particle size distribution of polyacrylate particles prepared via CEP and CEP + HD. (**a**) MP under different homogenizer pressure (**b**) and pass number (**c**). CEP denotes conventional emulsion polymerization, CEP + HD represents the emulsion containing HD synthesized by CEP, MP (m, n) signifies miniemulsion polymerization through homogenizer pressures of m, and pass number of n.

Figure 4 shows the particle size and particle size distribution (PSD) determined via DLS. Polyacrylate emulsion polymerized through CEP, CEP + HD, and MP all yielded unimodal PSDs because three polymerization techniques were carried out in semi-continuous mode instead of batch mode^21^. The addition of HD to CEP (CEP + HD) caused increase of the average particle size with respect to CEP. For CEP + HD, most of the particles were formed by homogeneous nucleation because the addition of HD does not form a stable miniemulsion with ordinary stirring equipment^28^. HD was hydrophobic enough to be distributed uniformly within monomer droplets at the beginning of polymerization^29^. As polymerization progressed, the monomers would gradually deliver from monomer droplets into the micelles. The hydrophobicity of HD made it difficult to transfer from the aqueous phase to polymerization loci. Therefore, the emulsion system will contain HD droplets in addition to polymer particles. HD droplet increased the viscosity of the dispersed phase and restrained free radical transfer from the aqueous phase to nucleated monomer-swollen micelles^30^. It was thus less prone to bi-radical termination in particles and led to bigger particle size after the addition of HD.

All emulsions prepared by MP had narrower particle size distributions than CEP and CEP + HD. When the HD containing emulsions were homogenized using a high-pressure homogenizer, extremely stable monomer miniemulsions were obtained and the polymer particles were similar to those of monomer droplets in MP. The particle diameter reduced initially when homogenizing CEP + HD at 300 bar (from 172 nm to 144 nm) and then increased to 164 nm with increase the homogeneous pressure to 1200 bar. Floury reported that high pressure led to higher coalescence rate and hence bigger particles^31^. At a constant homogenous pressure of 900 bar, the average diameter of particles were nearly unchanged among different pass numbers. This might be because the pressurized miniemulsion exiting the homogenizer was placed in an ice-bath to mitigate temperature increase at the exit of the homogenization valve and the particle aggregate^32^.

### Glass-transition temperatures of isolated films

The glass-transition temperature, which was closely related to film-formation states of the CRU coating, was used as an indirect indicator to select the CRU coatings. As shown in Table 1, the addition of HD to CEP decreased the *T*_*g*_ of isolated films. *T*_*g*_ reduced further with increasing homogeneous pressure and pass number when HD-containing emulsions were homogenized with a high-pressure homogenizer. In CEP + HD, HD droplets were mainly dispersed in the exteriors of particles, which reduced interparticle polymer chain interactions. Capeletto (2016) reported that n-pentane dispersed into polymeric matrix reduced the *T*_*g*_ of isolated films^33^. In MP, the majority of HD was distributed in the interiors of particles and the proportions of HD in the interior of particles increased with homogenizer pressure and pass number. HD in the interior of particles reduced the intraparticle polymer chains interactions. The higher amount of HD in the interior of particles increased the mobility of polymer chains and reduced the *T*_*g*_ of the polymer more effectively^33^.

**Table 1:** The glass-transition temperatures and mechanical properties of the isolated films.

Sample Code	*T*_*g*_ (°C)	Tensile stress at break (MPa)	Elongation at break (%)	Young’s modulus (MPa)
CEP	6.38	4.66 ± 0.30 a	1013 ± 100 b	36.00 ± 4.86 c
CEP + HD	4.37	3.14 ± 0.57 a	1021 ± 70 b	37.50 ± 5.48 c
MP (300,3)	3.92	4.25 ± 0.04 a	1046 ± 122 b	36.03 ± 3.20 c
MP (600,3)	3.86	3.45 ± 0.08 a	1049 ± 107 b	35.68 ± 5.02 c
MP (900,3)	3.06	4.46 ± 0.71 a	1092 ± 221 b	39.83 ± 4.26 c
MP (1200,3)	2.49	2.94 ± 1.12 a	1051 ± 107 b	35.02 ± 2.38 c
MP (900,1)	3.45	3.80 ± 0.32 a	1214 ± 148 b	41.64 ± 3.81 c
MP (900,5)	2.42	2.51 ± 0.16 a	1208 ± 263 b	43.49 ± 1.28 c
MP (900,7)	1.49	4.11 ± 1.15 a	1091 ± 124 b	33.62 ± 6.56 c

Means with the same letter are not significantly different at p ≤ 0.05 level.

### Mechanical properties of isolated films

It is necessary to develop coatings that combine flexibility and strength to obtain CRU with the desired performance. Elongation at break is a measure of film flexibility, whereas tensile stress is a measure of film strength. Materials with high values of Young’s modulus are associated with high stiffness but low flexibility. Table 1 demonstrates the mechanical properties of isolated films. The values of tensile stress, elongation at break, and Young’s modulus were not statistically different among CEP, CEP + HD, and MP (P > 0.05). The addition of HD and the miniemulsion polymerization at different homogenous pressures and pass numbers had little ability to change the mechanical properties of the coating. The mechanical properties were directly related to the polymer cross-linking degree, and the cross-linking degree did not change obviously among CEP, CEP + HD, and MP^34^.

### Water absorption of isolated films

**Figure 5:**
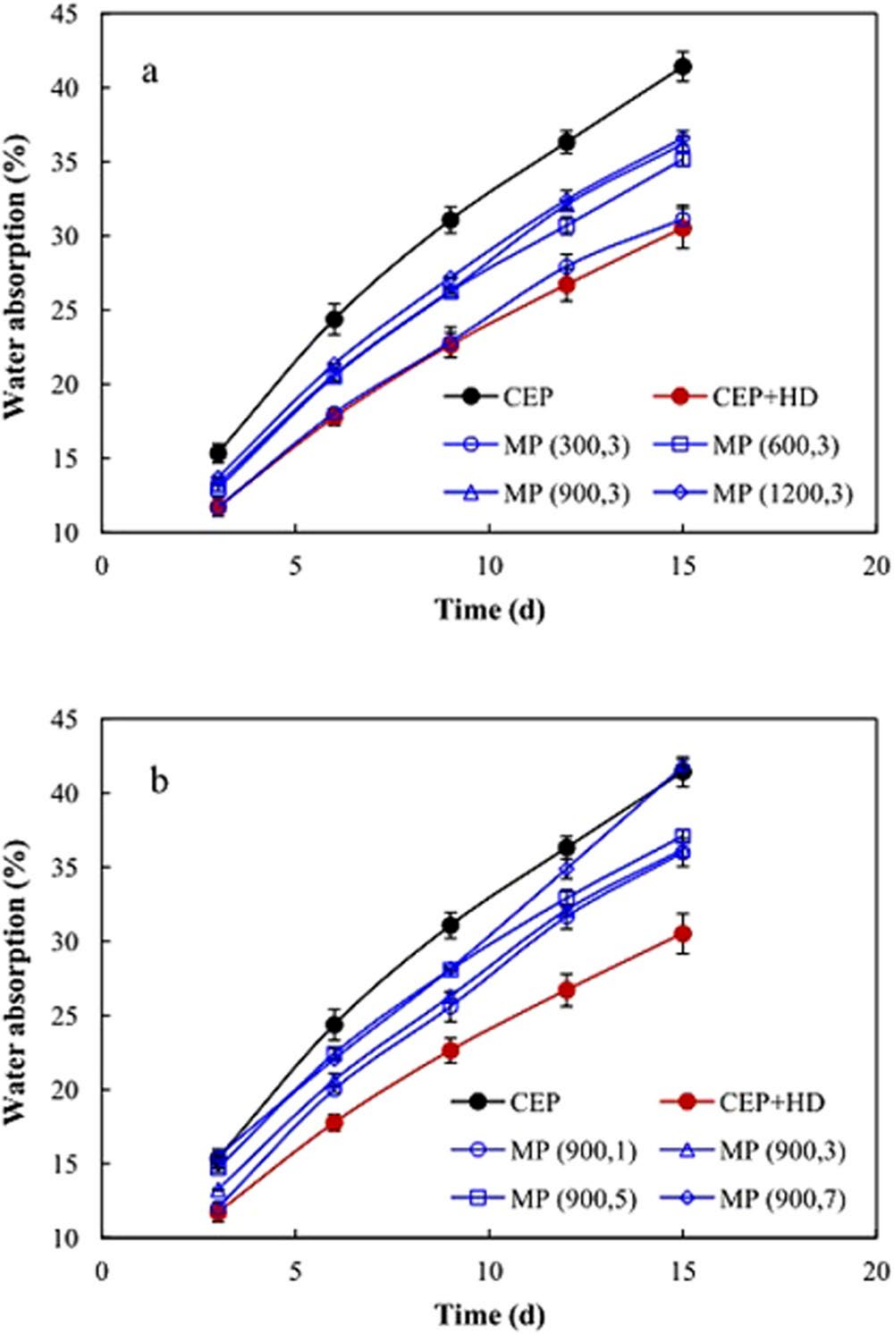
Water absorption of isolated films prepared via CEP, CEP + HD and MP under different homogenizer pressure (**a**) and pass number (**b**). CEP denotes conventional emulsion polymerization, CEP + HD represents the emulsion containing HD synthesized by CEP, MP (m, n) signifies miniemulsion polymerization through homogenizer pressures of m, and pass number of n.

**Figure 6:**
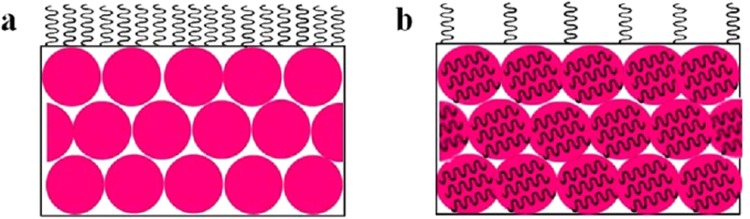
Schemes for the distribution of HD in the film of CEP + HD (**a**) and MP (**b**). CEP + HD represents the emulsion containing HD synthesized by CEP, MP signifies miniemulsion polymerization.

Water absorptions of isolated films were important evidence of water-resistance performance and lower water absorption indicated stronger water-resistance performance. As shown in Fig. 5, water absorption of CEP + HD and MP were lower than that of CEP due to the high hydrophobicity of HD. Pham *et al*. (2017) also reported that the hydrophobicity of polymer was enhanced by the addition of HD in oil droplets^29^. The reduction degree of CEP + HD was significantly more than that of MP. The water absorption of isolated films increased with homogenizer pressure (Fig. 5a) and pass number (Fig. 5b). The distribution of HD during film-forming process also played a key role in controlling the water-resistance performance of the coating. HD droplets distributed in the exteriors of particles can easily migrate to the surface of the coating and enhanced the water-resistance performance more effectively (Fig. 6a), while HD distributed in the interiors particles is difficult to migrate to the surface of the coating and the majority of HD still existed in the particles (Fig. 6b). The proportion of HD in the interior of particles increased with homogenizer pressure and pass number, thus the proportion of HD migrated to the coating surface decreased. Although HD was freely incorporated in the polyacrylate network, the hydrophobicity of HD made it difficult to rapidly dissolve in the release medium.

### FTIR-PAS of isolated films

**Figure 7:**
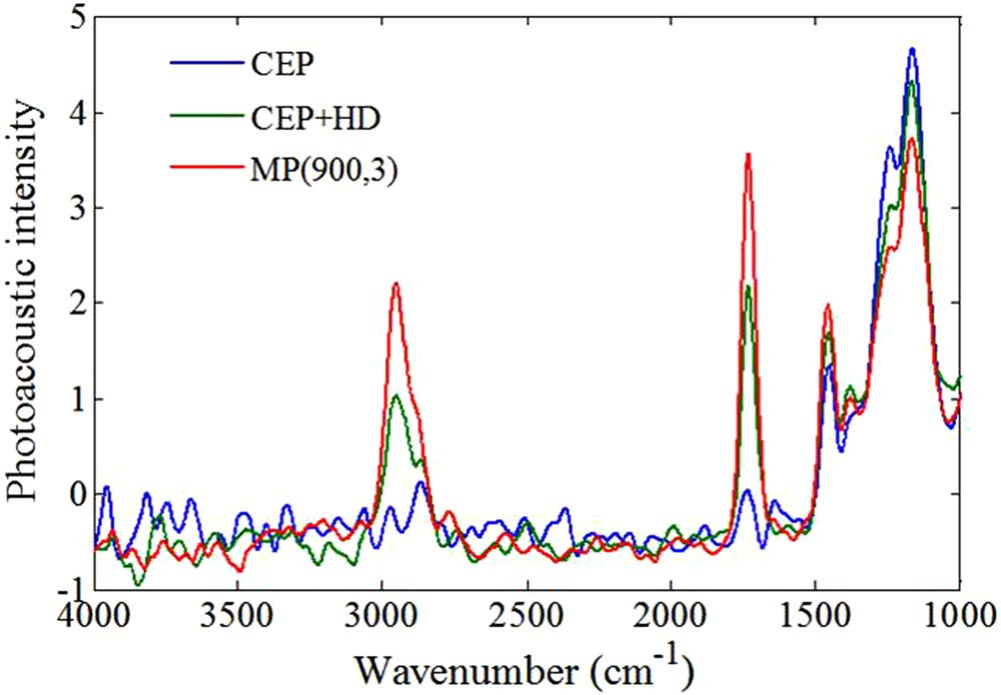
FTIR-PAS of CRU prepared with via CEP, CEP + HD and MP(900,3). CEP denotes conventional emulsion polymerization, CEP + HD represents the emulsion containing HD synthesized by CEP, MP (900,3) signifies miniemulsion polymerization through homogenizer pressures of 900 bar, and pass number of 3.

The FTIR-PAS of isolated films prepared with CEP, CEP + HD and MP (900,3) testified the distribution of HD during film-forming process (Fig. 7). The characteristic peaks of HD, the C–H stretching vibration at 2951 cm^−1^ and the C–H bending vibration at 1456 cm^−1^, showed a lower intensity in CEP + HD than MP(900,3). Spectral signals of internal films were obtained when the mirror velocity was set to 0.32 cm/s. It proved the majority of HD migrated to the surface of the films of CEP + HD, while most HD still retained inside the films of MP.

## Discussion

The final nutrient-release profiles of the CRU were directly related to the physicochemical properties of the coating, i.e., the water-resistance performance, glass-transition temperature, and mechanical properties. There was a close relationship between water-resistance performance of coating and nutrient release of CRU. The ability to insulate water from the hydrophobic surface of the coating improved the controlled-release ability of CRU. This was because slower water accumulation in the internal urea core reduced the transport medium of the nutrient. The strongest water-resistance performance of CEP + HD also permitted the slowest nutrient release of CRU. The nutrient release of CRU was also dependent on the glass-transition temperature of the coating. Low glass-transition temperature increased in the efficiency of particle coalescence when particles collided with each other and promoted the formation of a compact coating, which was beneficial in controlling nutrient release^32^. However, on the other hand, Low glass-transition temperature decreased the processability of CRU and destroyed the integrity of the coating because of the adhesion of fertilizer granules during preparation and storage. Incorporation of HD into CEP decreased the glass-transition temperature of the coating from 6 °C to 4 °C. The efficiency of coalescence between polymer particles was enhanced and a more compact coating was formed. When the glass-transition temperature was further decreased by MP, the CRU coating stuck together and the integrity of coating was destroyed when sticky CRU granules were separated. Stronger water-resistance performance and more compact coating gave CEP + HD slower nutrient release from the coated fertilizer.

To slow down the nutrient release of CRU coated with aqueous polyacrylate, the effects of hexadecane and polymerization technique on the properties of emulsions, films, and the resultant nutrient release profiles of CRU were investigated. The incorporation of hexadecane into polyacrylate strikingly delayed urea release from controlled release urea because of stronger water-resistance performance and more compact coating. The distribution of HD played an important role in controlling the glass-transition temperature and water-resistance performance of the coating, which were strongly correlated with the controlled-release profile of CRU. Most of HD was distributed in the interiors of particles for MP and exteriors of particles for CEP + HD. Exterior HD improved water resistance more effectively, while interior HD reduced glass-transition temperature more significantly. Further studies are needed to address optimization of the HD content to further slow nutrient release from CRU. Nutrient release from CRU was significantly retarded by adding HD during the production of conventional emulsion, but not by the miniemulsion polymerization technique.

## Materials and Methods

### Materials

Urea granules (46.6% of nitrogen and 2.00–4.75 mm in diameter) were provided by Shandong Luxi Fertilizer Co., Ltd. Methyl methacrylate (MMA, AR), n-butyl acrylate (BA, CP), and methacrylic acid (MAA, CP) were obtained from Nanjing Chemical Reagent Co., Ltd. Sodium dodecylbenzenesulfonate (SDBS, AR) was purchased from Chengdu Kelong Chemical Reagent Co., Ltd. Nonyl phenyl polyoxyethylene ether-10 (OP-10, CP) was received from Hebei Xingtai Kewang Auxiliary Agent Co., Ltd. Potassium persulfate (KPS, CP) and hexadecane (HD, CP) were supplied by Sinopharm Chemical Reagent Co., Ltd. All reagents were used as received. Deionized water was used to prepare all emulsions and solutions.

### Preparation of polyacrylate emulsions

Polyacrylate emulsions were synthesized using CEP, CEP + HD, and MP. The CEP was carried out in a 1 L three-necked flask equipped with a mechanical stirrer, reflux condenser, and an isobaric funnel. SDBS (4.12 g) and OP-10 (8.24 g) were dissolved in deionized water (250 mL) for the aqueous phase; BA (110 g), MMA (90 g), and MAA (5 g) were mixed for the oil phase. Both phases were vigorously stirred for 30 min and 25 wt% of the oil-water mixture in the flask was used as initial charge. The temperature was raised to 80 °C till the end of polymerization. The initiator solution (52 mL, 0.01 g/mL, KPS) and the remainder of the oil-water mixture were fed alternately in four doses over 3 h, and polymerization was conducted under an air atmosphere for an additional 3 h. For MP, 8.24 g of HD—used as co-stabilizer—was mixed with acrylate monomer to form the organic phase. Both phases were mixed and stirred vigorously for 30 min to generate the pre-emulsion. The pre-emulsion was homogenized with an APV Lab Series Homogenizer (APV-2000, SPX FLOW, Germany) at desired homogenization pressure and cycle numbers. During homogenization, the vessel containing the emulsion was immersed in an ice-bath to mitigate temperature increase at the exit of the homogenization valve. The resultant miniemulsion was transferred to a flask and polymerization was performed as in CEP. Seven miniemulsions were prepared and labeled as MP (m, n), signifying homogenizer pressure of m, and pass number n. HD might influence polyacrylate properties and could possibly create bias in the comparison of properties between CEP and MP. Thus CEP + HD was synthesized in the same manner as CEP, except that 8.24 g of HD was added to the CEP production recipes. Solid contents of polyacrylate emulsions synthesized via CEP, CEP + HD, and MP were all about 40%. Isolated films were prepared using a casting method, where an emulsion was cast on a nonstick substrate and water was evaporated completely in an oven at 60 °C for 72 h.

### Characterization of emulsions and isolated films

The particle size (average diameter (*D*_*mean*_)) and particle size distributions (PSD) were measured using a dynamic light scattering (DLS) analyzer (Nano ZS90, Malvern Instruments). Before measurements, the emulsions were prepared with the concentration of 0.01 g/mL. A He-Ne laser with a wavelength of 633 nm was irradiated to the polymer emulsion, and the scattering light was detected at the angle of 90° at 25 °C. The measured autocorrelation functions were analyzed with the Zetasizer Software 6.32 software. The transmission electron microscopy (TEM) observations were carried out on a JEOL-2000EX transmission electron microscope equipped with a double-tilt holder operating at 160 kV. The polyacrylate emulsions were diluted with water, dropped on a carbon coated copper grid, and then were negatively stained with an aqueous solution of 2 wt% phosphotungsticacid before examination. Approximately 10 mg of isolated film was weighed and differential scanning calorimetry (DSC, Perkin-ElemrPyris1, USA) was taken at heating rate of 20 °C/min. Thermal behaviors of the samples were examined under nitrogen between −100 °C and 150 °C. The glass transition temperature (*T*_*g*_) was taken at the onset of the corresponding heat capacity jump. Two successive scans were made for each sample. All calculations were performed on the second heating cycle. The mechanical properties of isolated films were obtained using a universal testing machine (CMT 5254, Shenzhen SANS Testing Machine Co., Ltd., China) according to procedures outlined in ASTM D 638–03, with five replicates. A dumbbell shaped die (type A2) was cut. The initial gauge length was 10 mm and the measurement speed was 200 mm/min. Tensile stress is presented relative to the original cross-sectional area of the sample. Elongation at break is defined as the elongation ratio with respect to the initial gauge length when the film broke. The Young’s modulus is calculated from the first slope of the stress-strain curve using least-squares best fitting. Water absorptions of isolate films were determined by the following procedure^35^: the weighed film (*W*_*f1*_) was immersed in deionized water at 25 °C for 15 d. The swollen films were removed from water every three days and the surface water was wiped off with a piece of filter paper to determine the weight (*W*_*f2*_). Water absorption was defined as (*W*_*f2*_ − *W*_*f1*_) × 100/*W*_*f1*_. A FTIR spectrometer (Nicolet 380, USA) equipped with photoacoustic accessory (Model 300, MTEC, USA) was used for the determination of the spectra of CRU coated with pure polyacrylate and composite polyacrylate. The spectra were recorded in wave number range of 650−4000 cm^−1^, and the mirror velocity was set to 0.32 cm/s, 32 successive scans were conducted with a resolution of 4 cm^−1^.

### Preparation of CRU

Fertilizer granules were coated in a Wurster fluidized bed equipment (LDP-3, Changzhou Jiafa Granulation Drying Equipment Co., Ltd., China). For typical coating experiment, 400 g of urea granules was loaded into the bed and spouted with warm air. The emulsion was sprayed uniformly to the constantly fluidized urea granules, which were preheated at 45 °C for 10 min. A peristaltic pump was used to pump the emulsion (100 g) to the atomizing nozzle located at the bottom of the central orifice of the fluidized bed. The process parameters were: product temperature 45 °C, spray rate 2.5 g/min, and atomization pressure 0.1 MPa. The coated granules were tray-dried in an oven at 60 °C for 24 h before further evaluation. Coated urea granules consisted of urea cores surrounded by polyacrylate coating with thickness of ~100 μm and coating content 9.1% of the total weight of coated urea granule.

### Nutrient release profiles of CRU

Here, 5 g of polymer-coated fertilizer was immersed in 100 mL of deionized water at 25 °C. Urea solution (100 mL) was removed after 1, 3, 5, 7, 10, 14, and 28 d and replaced with 100 mL of deionized water for each of three replicates. Urea content was evaluated by the para-dimethylaminobenzaldehyde colorimetric method (Epoch microplate spectrophotometer, Biotek). On day 28 of release, the CRU was ground to determine the content of residual nutrient. The nutrient release profiles were estimated as the cumulative dissolution versus time.
